# A Comparative Study Between Arm Intravenous Regional Anesthesia Versus Forearm Intravenous Regional Anesthesia in Patients Undergoing Hand and Wrist Surgery

**DOI:** 10.5812/aapm-164630

**Published:** 2025-08-25

**Authors:** Elsayed Kamel Youssef, Mohamed Hossam Eldin Hamdy Shokeir, Mohammed M Maarouf, Hany Magdy Fahim, Hany V. Zaki

**Affiliations:** 1 Department of Anesthesia, Intensive Care, and Pain Management, Faculty of Medicine, Ain Shams University, Cairo, Egypt

**Keywords:** Regional Anesthesia, Anesthesia, Intravenous, Forearm, Hand Surgery, Wrist, Analgesia

## Abstract

**Background:**

Intravenous regional anesthesia (IVRA) is a widely used technique for hand and wrist surgeries. However, conventional upper arm IVRA requires higher anesthetic doses, which increases the risk of systemic toxicity. Forearm IVRA offers potential advantages, including lower anesthetic requirements and improved tourniquet tolerance.

**Objectives:**

The study aims to compare the efficacy, analgesic effectiveness, and safety of forearm versus upper arm IVRA in elective hand and wrist surgeries.

**Methods:**

In this prospective, randomized, open-label clinical trial, 140 adult patients, classified as American Society of Anesthesiologists (ASA) physical status I - II and aged 21 - 65 years, scheduled for elective hand or wrist surgery at Ain Shams University Hospitals, were randomized into two equal groups: Upper arm IVRA and forearm IVRA. The outcomes measured included block success, onset of analgesia, tourniquet pain-free duration, pain scores, rescue analgesia requirements, and patient and surgeon satisfaction.

**Results:**

Block success rates were similar between the forearm and upper arm IVRA groups (94.3% vs. 91.4%, P = 0.512). Forearm IVRA demonstrated a significantly longer tourniquet pain-free duration (45.7 ± 4.6 vs. 43.2 ± 4.7 minutes, P = 0.002) and a longer time to the first postoperative analgesic request (8.9 ± 0.9 vs. 5.8 ± 1.0 hours, P < 0.001), with lower 24-hour nalbuphine consumption (11.4 ± 4.2 vs. 28.7 ± 3.4 mg, P < 0.001). Patient satisfaction was higher in the forearm group (P < 0.001), while surgeon satisfaction did not differ significantly (P = 0.145)

**Conclusions:**

Forearm IVRA is an effective and safe alternative to upper arm IVRA for hand and wrist surgeries, offering superior tourniquet tolerance, prolonged analgesia, reduced opioid requirements, and higher patient satisfaction

## 1. Background

Hand and wrist surgeries are among the most frequently performed procedures in orthopedic and plastic surgery. These operations often require effective anesthesia techniques that ensure patient comfort, provide a bloodless surgical field, and allow for rapid recovery (1). Although general anesthesia is an option, regional techniques — such as intravenous regional anesthesia (IVRA) — are often preferred due to their cost-effectiveness, technical simplicity, and ability to provide excellent intraoperative conditions with minimal systemic effects (2-4).

The traditional Bier’s block technique, introduced by August Bier in 1908, involves applying a tourniquet on the upper arm after limb exsanguination to isolate the local anesthetic within the limb’s vasculature (5, 6). This method reliably produces surgical anesthesia for hand procedures. However, a notable drawback is the relatively high dose of local anesthetic required for upper arm IVRA, which raises the risk of systemic toxicity after tourniquet deflation. Additionally, upper arm tourniquets can lead to significant discomfort and ischemic pain during the procedure, sometimes necessitating additional sedation or conversion to general anesthesia (7, 8).

To overcome these limitations, the use of forearm IVRA — also known as the mini-Bier’s block — has been proposed. This technique involves placing the tourniquet on the forearm instead of the upper arm, reducing the vascular bed volume and allowing for lower doses of local anesthetic (6, 9). Emerging studies suggest that forearm IVRA may offer faster onset, improved tourniquet tolerance, and comparable or superior analgesic efficacy, all while reducing the risk of systemic toxicity (8, 10, 11). Despite these promising findings, evidence from high-quality comparative trials remains limited. The relative advantages of forearm versus upper arm IVRA continue to be debated, particularly with regard to pain control, block success, and postoperative recovery.

Therefore, a well-designed randomized controlled trial is warranted to clarify the clinical utility of forearm IVRA and determine whether its theoretical benefits translate into improved patient outcomes. We hypothesize that forearm IVRA provides equivalent or superior analgesic efficacy compared to conventional upper arm IVRA, with faster onset, better tourniquet tolerance, and reduced anesthetic requirements, ultimately enhancing patient and surgeon satisfaction.

## 2. Objectives

The objective is to compare the efficacy, safety, and overall outcomes of arm versus forearm IVRA in patients undergoing elective hand and wrist surgeries

## 3. Methods

### 3.1. Study Design and Setting

This prospective, randomized, open-label comparative clinical trial was conducted on 140 adult patients scheduled for elective hand and wrist surgery under IVRA at the Anesthesia, Intensive Care, and Pain Management Department, Ain Shams University Hospitals, over a one-year period from January to December 2023.

### 3.2. Eligibility Criteria

#### 3.2.1. Inclusion Criteria

Patients were eligible for inclusion if they were aged between 21 and 65 years, of either sex, classified as American Society of Anesthesiologists (ASA) physical status I or II, and scheduled for elective hand or wrist surgery under IVRA. Eligibility was confirmed following a preoperative assessment by the attending anesthesiologist.

#### 3.2.2. Non-inclusion Criteria

Patients who declined to participate in the study or were unable to provide informed consent were not included. Additionally, patients with incomplete clinical data or who were lost to follow-up were excluded from the final analysis.

#### 3.2.3. Exclusion Criteria

Exclusion criteria included patients with a Body Mass Index (BMI) ≥ 40 kg/m², ASA physical status III or higher, or those undergoing bilateral hand surgeries. Patients with known allergies to local anesthetics, local site infections, pre-existing myopathy or neuropathy in the operative limb, chronic analgesic abuse, or significant cognitive dysfunction were also excluded.

### 3.3. Sample Size and Randomization

Using PASS 15 software for sample size estimation and based on prior findings from Dekoninck et al. (8), which demonstrated greater analgesic effectiveness of forearm IVRA compared to conventional upper arm IVRA, we assumed a medium effect size (Cohen’s d = 0.5) for differences in analgesic scores between the two groups. To achieve 80% power at a 5% alpha error and accounting for a 10% potential dropout rate, a minimum of 70 patients per group (140 patients in total) was calculated as necessary. Randomization was done by a computer-generated table of random numbers, and group assignments were concealed in sequentially numbered opaque envelopes opened just before the procedure.

### 3.4. Preoperative Preparation and Monitoring

Upon admission to the operating room, patients were positioned supine, and standard monitoring protocols were initiated, encompassing non-invasive arterial pressure measurement, electrocardiographic assessment, and peripheral oxygen saturation monitoring. An IV line was established in the contralateral arm for fluid and medication administration. Intravenous paracetamol at a dose of 15 mg/kg (maximum 1 g) was administered as preemptive analgesia (8, 12). NSAIDs and dexamethasone were avoided preoperatively and intraoperatively to standardize analgesic regimens.

### 3.5. Interventions

In patients assigned to the upper arm IVRA group, a double pneumatic tourniquet was positioned proximally above the elbow following limb exsanguination with an Esmarch bandage. The proximal cuff was inflated to 250 mmHg, and 40 mL of 0.5% lidocaine was gradually administered via a 22-gauge cannula inserted into a dorsal vein of the hand. To minimize the risk of systemic local anesthetic toxicity, the tourniquet remained inflated for at least 60 minutes. For the forearm IVRA group, the tourniquet was applied approximately 5 cm distal to the medial epicondyle and similarly inflated after exsanguination; 25 mL of 0.5% lidocaine was then injected. The tourniquet duration was maintained for no less than 45 minutes. In both groups, distal circulatory arrest was verified prior to anesthetic administration to ensure adequate vascular isolation.

### 3.6. Intraoperative Management and Rescue Protocols

Patients were continuously monitored for hemodynamic stability. Hypotension, defined as a decrease in SBP > 20% from baseline, was treated with IV ephedrine (10 - 30 mg) titrated to response. Bradycardia associated with signs of hypoperfusion was managed with IV atropine (0.5 mg, repeated as needed up to 3 mg). Supplemental oxygen was provided to maintain peripheral oxygen saturation (SpO₂) above 94%. Intraoperative pain was assessed every 5 minutes using an 11-point Numeric Rating Scale (NRS) (13), where 0 represented no pain and 10 the worst imaginable pain. Rescue analgesia with fentanyl (0.5 - 1 mcg/kg) was administered if pain reached an NRS score of 5 or higher. Conversion to general anesthesia was considered if pain remained uncontrolled despite rescue analgesia.

### 3.7. Postoperative Care and Follow-up

Patients were monitored in the post-anesthesia care unit (PACU) and followed for 24 hours postoperatively. Pain scores were recorded every two hours using the NRS. Rescue analgesia was administered with intravenous nalbuphine (10 mg) if NRS ≥ 5, followed by regular doses of intravenous paracetamol (1 g every 6 hours) and NSAIDs (every 8 hours) for pain control during the first 24 hours. Time to first rescue analgesia and total nalbuphine consumption were recorded. Patient satisfaction with the anesthesia technique was evaluated on postoperative day 1 using a 7-point Likert scale, while surgeons assessed the surgical field quality with a 5-point Likert scale.

### 3.8. Outcomes

Primary outcomes included block quality (graded I - IV) and overall block success (complete or incomplete analgesia). Secondary outcomes comprised onset time of analgesia, tourniquet pain-free duration, total tourniquet and surgery times, intraoperative and postoperative pain scores, rescue analgesia requirements, and patient and surgeon satisfaction scores.

### 3.9. Statistical Analysis

Statistical analysis and data handling were performed using IBM SPSS Statistics version 27 (IBM Corp., Armonk, NY, USA). The distribution of continuous variables was evaluated through the Shapiro-Wilk test and graphical methods. Depending on the distribution pattern, continuous variables were expressed either as mean ± standard deviation for normally distributed data or as median and range for skewed data. Categorical variables were presented as frequencies and percentages. Intergroup comparisons of continuous variables were conducted using the independent samples *t*-test for parametric data and the Mann-Whitney U test for non-parametric data. Categorical variables were analyzed using either the chi-square test or Fisher’s exact test as appropriate. A two-tailed P-value < 0.05 was considered statistically significant.

## 4. Results

A total of 159 cases were initially screened for eligibility in this trial, of which 19 were excluded for not meeting the inclusion criteria. The remaining 140 participants were randomly allocated into two equal groups. All enrolled cases were subsequently monitored and included in the statistical analysis.

### 4.1. Demographics and Baseline Characteristics

**Figure 1.**
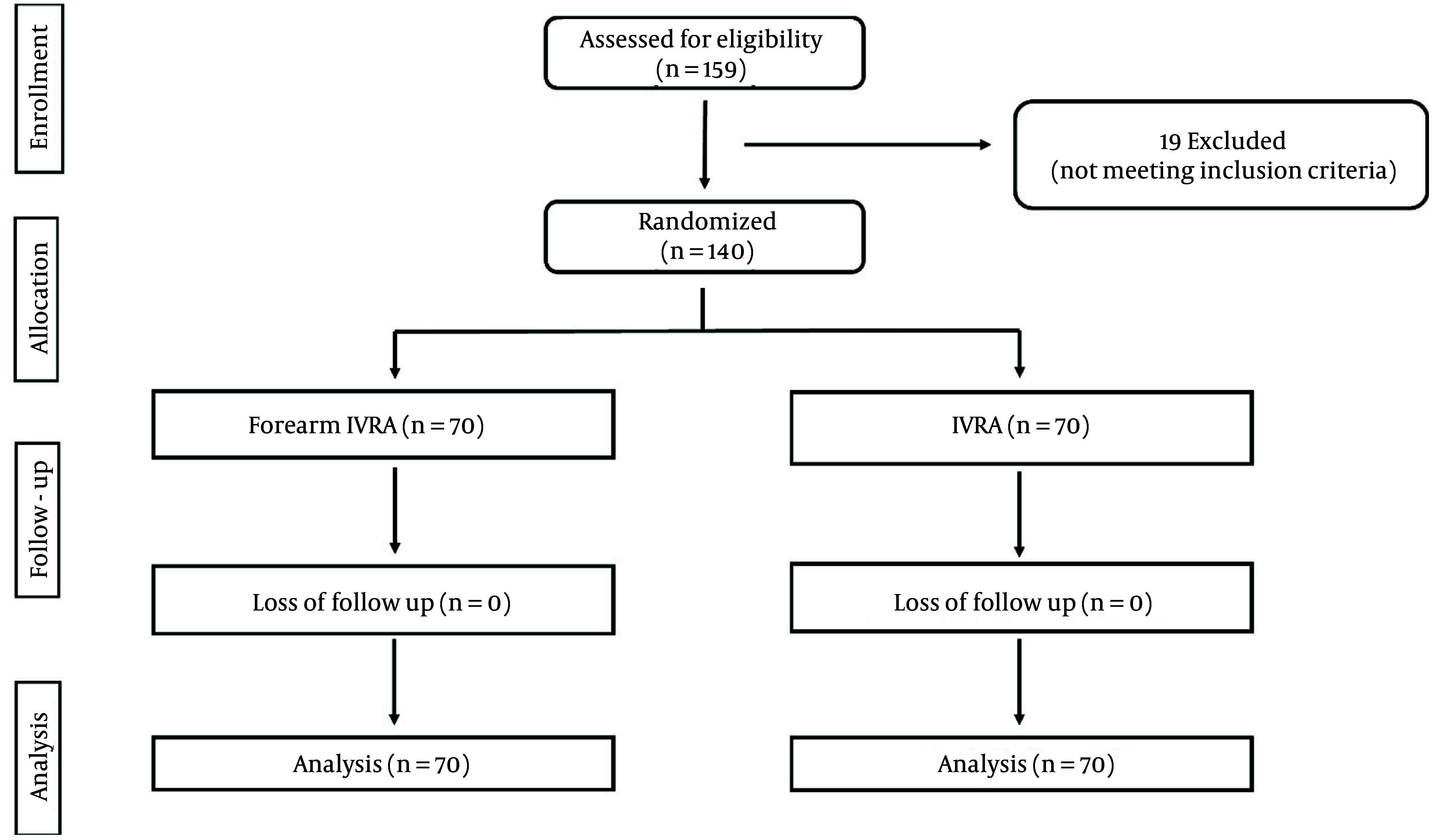
Consort flow diagram of the enrolled patients

Age (P = 0.239), gender distribution (P = 0.730), BMI (P = 0.629), and ASA classification (P = 0.387) did not significantly differ between the forearm and arm IVRA groups (Figure 1). 

### 4.2. Block Quality and Analgesia

Block quality grades (P = 0.770), overall block success (P = 0.512), and onset of analgesia (P = 0.077) showed no notable variations between the groups (Table 1). Tourniquet pain-free duration was notably longer in the forearm IVRA group compared to the arm IVRA group (45.7 ± 4.6 vs. 43.2 ± 4.7 min, P = 0.002). In contrast, total tourniquet duration (P = 0.193), surgery duration (P = 0.132), and operating room time (P = 0.189) did not show notable variations between the groups (Table 2). 

**Table 1.** General Characteristics, Block Quality, and Analgesic Effectiveness of the Studied Groups (N = 70) ^a^

General Characteristics	Forearm IVRA	IVRA	P-Value
**Age (y)**	34.6 ± 6.0	33.3 ± 6.6	0.239
**Gender**			0.73
Male	41 (58.6)	43 (61.4)	
Female	29 (41.4)	27 (38.6)	
**BMI (kg/m²)**	28.5 ± 3.2	28.8 ± 3.7	0.629
**ASA**			0.387
I	45 (64.3)	40 (57.1)	
II	25 (35.7)	30 (42.9)	
**Block quality grades**			0.77
Grade I	51 (72.9)	48 (68.6)	
Grade II	15 (21.4)	16 (22.9)	
Grade III	4 (5.7)	6 (8.6)	
**Overall block success**			0.512
Complete	66 (94.3)	64 (91.4)	
Incomplete	4 (5.7)	6 (8.6)	
**Onset of analgesia (min)**	7.8 ± 1.3	8.2 ± 1.2	0.077

Abbreviations: IVRA, intravenous regional anesthesia; BMI, Body Mass Index; ASA, American Society of Anesthesiologists.

^a^ Values are presented as No (%) or mean ± SD.

**Table 2.** Tourniquet and Procedural Times, and Rescue Analgesia Requirements Between the Studied Groups (N = 70) ^a^

Variables	Forearm IVRA	IVRA	P-Value
**Tourniquet pain-free duration (min)**	45.7 ± 4.6	43.2 ± 4.7	0.002 ^b^
**Total tourniquet duration (min)**	47.0 ± 4.5	48.0 ± 4.9	0.193
**Surgery duration (min)**	54.9 ± 7.1	53.0 ± 7.6	0.132
**Operating room time (min)**	86.9 ± 7.2	85.3 ± 7.7	0.189
**Rescue analgesia**	37 (52.9)	46 (65.7)	0.122
**First analgesic request (h)**	8.9 ± 0.9	5.8 ± 1.0	< 0.001^b^
**24-hrs Nalbuphine consumption (mg)**	11.4 ± 4.2	28.7 ± 3.4	< 0.001 ^b^

Abbreviations: n, number; IVRA, intravenous regional anesthesia; SD, standard deviation, mg, milligrams.

^a^ Values are presented as No (%) or mean ± SD.

^b^ Significant P-value.

### 4.3. Analgesic Requirements and Pain Scores

Patients in the forearm IVRA group had a significantly longer time to first analgesic request (8.9 ± 0.9 vs. 5.8 ± 1.0 hours, P < 0.001) and substantially lower total 24-hour nalbuphine consumption (11.4 ± 4.2 vs. 28.7 ± 3.4 mg, P < 0.001) compared to the arm IVRA group. The proportion of patients requiring rescue analgesia did not differ substantially between groups (52.9% vs. 65.7%, P = 0.122) (Table 2). 

**Figure 2.**
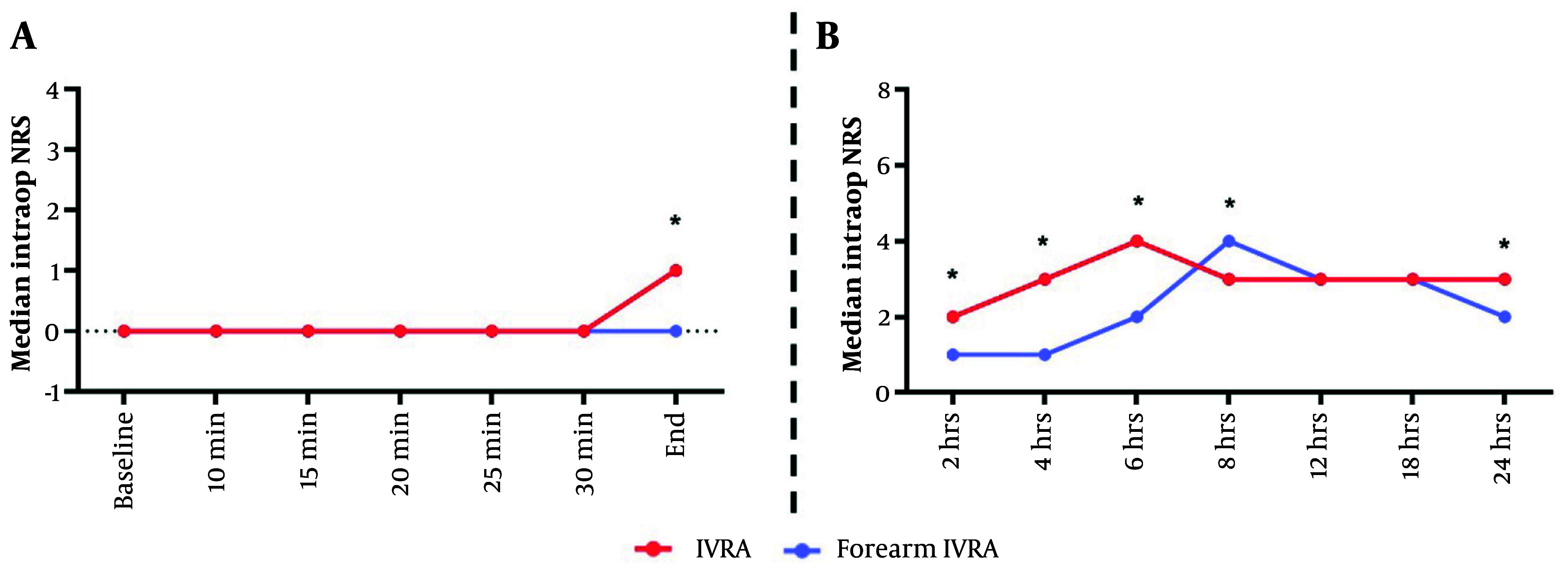
Median A, intra-operative; and B, postoperative pain scores (NRS) between the study groups

Intraoperative pain at the end of surgery was substantially higher in the forearm IVRA group compared to the arm IVRA group, with median (range) scores of 1 (0 - 3) versus 0 (0 - 1), respectively (P < 0.001). Postoperatively, the forearm IVRA group exhibited significantly higher pain scores at 2 hours [median 2 (0 - 3) vs. 1 (0 - 2), P < 0.001], 4 hours [3 (1 - 6) vs. 1 (0 - 2), P < 0.001], 6 hours [4 (1 - 6) vs. 2 (1 - 3), P < 0.001], 8 hours [3 (3 - 5) vs. 4 (1 - 6), P = 0.019], and 24 hours [3 (1 - 4) vs. 2 (2 - 3), P = 0.020]. Intraoperative pain at minute-25 (P = 0.057) and minute-30 (P = 0.055), as well as postoperative pain at 12 hours (P = 0.133) and 18 hours (P = 0.152), did not show notable changes between the groups (Figure 2A and B).

### 4.4. Satisfaction Scores

**Figure 3.**
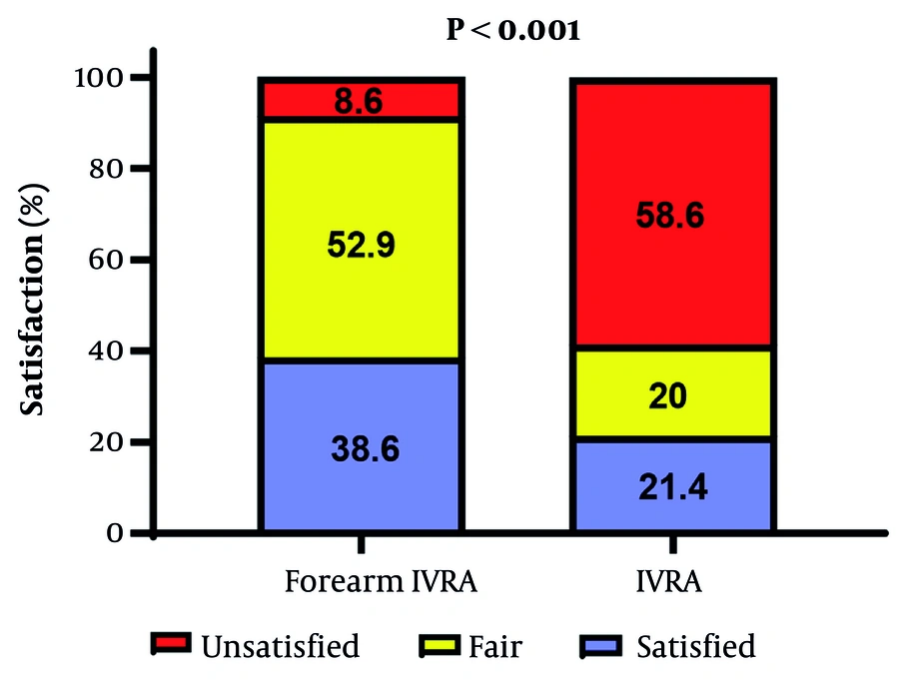
Patients’ satisfaction between the study groups

Patients receiving forearm IVRA exhibited significantly higher satisfaction, with 38.6% reporting being satisfied and 52.9% reporting fair satisfaction, compared to 21.4% satisfied and 20.0% fair in the arm IVRA group (P < 0.001) (Figure 3). In contrast, surgeons’ satisfaction did not significantly differ between groups, with 94.3% satisfied and 5.7% fair in the forearm IVRA group versus 87.1% satisfied and 12.9% fair in the arm IVRA group (P = 0.145).

## 5. Discussion

In this trial, we compared the efficacy, analgesic effectiveness, and safety of forearm versus conventional upper arm IVRA in patients undergoing elective hand and wrist surgeries. Our findings demonstrated that both techniques achieved comparable rates of complete block success, with no significant differences in block quality or onset time of analgesia between the groups. However, the forearm IVRA group exhibited a significantly longer tourniquet pain-free duration and higher patient satisfaction scores, despite experiencing slightly higher intraoperative and early postoperative pain scores. These findings align with those reported in the literature.

In their systematic review and meta-analysis, Dekoninck et al. (8) concluded that forearm IVRA is equally effective as upper arm IVRA in providing surgical anesthesia, even with reduced, non-toxic doses of local anesthetic. They also emphasized its advantages, including faster sensory block onset, improved tourniquet tolerance with less ischemic pain, and minimal sedation requirements — all without compromising safety or block success. Their pooled analysis across 383 patients showed a 99.5% success rate, with only a single mild case of systemic toxicity.

Supporting this, Singh et al. (11) conducted a randomized trial comparing forearm and upper arm IVRA using a combination of lidocaine and ketorolac. They used exactly half the dose in the forearm group (1.5 mg/kg lidocaine + 0.15 mg/kg ketorolac) compared to the upper arm group (3 mg/kg lidocaine + 0.3 mg/kg ketorolac). Surgical anesthesia was rated as excellent or good in 95% of forearm cases and 100% of upper arm cases. Both groups demonstrated similar onset and regression of sensory block, with comparable analgesic requirements and 24-hour pain scores.

Further supporting evidence comes from Chiao et al. (14), who found that forearm IVRA significantly reduced tourniquet pain and sedation needs. In their randomized trial, patients in the forearm group required markedly less fentanyl (30 µg vs. 104 µg) and fewer required deep sedation with propofol (1 vs. 22 patients). Notably, 19 patients in the forearm group were discharged directly without requiring PACU recovery, compared to none in the upper arm group.

Similarly, Chong et al. (15) compared the two techniques in the setting of distal radius fracture manipulation. Both methods provided effective anesthesia, but the forearm technique was favored for its faster sensory block onset and lower anesthetic volume requirements — advantages that are particularly relevant in outpatient and resource-limited settings. The prolonged tourniquet tolerance observed in our study among the forearm group also mirrors findings by Karalezli et al. (16), who demonstrated improved patient comfort when the tourniquet was applied to the forearm. Their study of 120 patients reported a rapid mean sensory block onset time of 4.5 minutes, with no observed local or systemic complications, supporting the safety and efficacy of this modified approach.

However, not all findings in the literature are entirely consistent. For instance, Nijs et al. (5) conducted a non-inferiority trial and failed to confirm that forearm IVRA alone could reliably provide surgical block without additional rescue analgesia. Nonetheless, they did confirm non-inferiority in avoiding general anesthesia and noted less tourniquet-related discomfort in the forearm group. These discrepancies may be attributed to differences in anesthetic protocols, outcome definitions, or study designs.

While the forearm IVRA group in the current study showed slightly higher intraoperative and early postoperative pain scores, these did not translate into greater opioid consumption or patient dissatisfaction. This suggests that patients may consider the temporary discomfort an acceptable trade-off for the longer-lasting analgesia and reduced opioid need. Still, adequate patient counseling and tourniquet management strategies are essential to optimize comfort and cooperation during surgery.

A key strength of this study is the clear demonstration that forearm IVRA allowed for significantly lower total lidocaine doses without compromising block success. This finding is clinically relevant because systemic local anesthetic toxicity remains a major concern in upper arm IVRA, particularly in elderly or low-weight patients. Reducing the anesthetic volume not only minimizes this risk but also promotes faster recovery and earlier hospital discharge.

Interestingly, despite superior analgesic outcomes with forearm IVRA, intraoperative pain scores at the end of surgery and early postoperative periods were slightly higher compared to arm IVRA. One possible explanation is that the shorter distance between the forearm tourniquet and the surgical field may result in earlier onset of tourniquet-related discomfort or more rapid washout of the anesthetic upon tourniquet deflation. However, these transient differences did not translate into greater opioid consumption, suggesting they were clinically insignificant.

Surgeon satisfaction scores were high in both groups, without significant differences. This observation indicates that forearm IVRA does not compromise the quality of the surgical field, a key consideration for procedures requiring delicate dissection or microvascular techniques. From a practical standpoint, the increased patient satisfaction with forearm IVRA is likely attributable to reduced tourniquet pain and lower postoperative analgesic requirements, enhancing overall patient experience. These findings suggest that adopting forearm IVRA as a standard technique for appropriate hand and wrist procedures may improve perioperative outcomes and patient-centered care.

Although Volkmar et al. (10) reported similar results regarding the safety and analgesic effectiveness of forearm compared to upper arm IVRA, they found no significant difference in patient satisfaction between the two techniques, unlike our study. This discrepancy may be justified by our longer tourniquet pain-free duration and prolonged postoperative analgesia in the forearm group, which likely contributed to improved patient comfort and satisfaction.

Despite these strengths, the study has limitations. First, the trial was conducted at a single tertiary center, which may limit the generalizability of the results to other practice settings or patient populations. Second, the study was open-label and no blinding was implemented due to the nature of the intervention, which may introduce observer or performance bias, particularly in subjective outcomes such as pain scores and patient satisfaction. Third, the study excluded patients with ASA III or higher and those with extreme BMI, who may represent important subgroups at greater risk of complications. Fourth, long-term outcomes beyond 24 hours were not assessed, precluding conclusions about prolonged analgesia or late complications. Future multicenter trials including a broader patient population, blinding where feasible, and extended follow-up are needed to validate these findings.

### 5.1. Conclusions

This study demonstrated that forearm IVRA is an effective and safe alternative to conventional upper arm IVRA for elective hand and wrist surgeries, offering longer tourniquet tolerance and higher patient satisfaction with reduced postoperative opioid consumption. It can be considered as a preferred technique in suitable patients, although further multicenter studies with longer follow-up are warranted to confirm these benefits and assess potential late complications.

## Data Availability

The dataset presented in the study is available on request from the corresponding author during submission or after publication.

## Clinical Trial Registration Code:

NCT06448845.
